# The Beneficial Roles of SIRT1 in Drug-Induced Liver Injury

**DOI:** 10.1155/2019/8506195

**Published:** 2019-07-01

**Authors:** Tingdong Yan, Jinlong Huang, Muhammad Farrukh Nisar, Chunpeng Wan, Weifeng Huang

**Affiliations:** ^1^Department of Pharmacology, School of Pharmacy, Nantong University, Nantong, 226001, Jiangsu, China; ^2^The Institute of Infection and Inflammation, Department of Microbiology and Immunology, Medical College, China Three Gorges University, Yichang, Hubei 443002, China; ^3^Department of Physiology and Biochemistry, Cholistan University of Veterinary and Animal Sciences (CUVAS), Bahawalpur, 63100, Pakistan; ^4^Jiangxi Key Laboratory for Postharvest Technology and Nondestructive Testing of Fruits & Vegetables, Collaborative Innovation Center of Post-Harvest Key Technology and Quality Safety of Fruits and Vegetables, College of Agronomy, Jiangxi Agricultural University, Nanchang 330045, China

## Abstract

Drug-induced liver injury (DILI) is a major cause of acute liver failure (ALF) as a result of accumulated drugs in the human body metabolized into toxic agents and helps generate heavy oxidative stress, inflammation, and apoptosis, which induces necrosis in hepatocytes and ultimately damages the liver. Sirtuin 1 (SIRT1) is said to have multiple vital roles in cell proliferation, aging, and antistress systems of the human body. The levels of SIRT1 and its activation precisely modulate its critical role in the interaction between multiple step procedures of DILI. The nuclear factor kappa-light-chain-enhancer of activated B cell- (NF-*κ*B-) mediated inflammation signaling pathway, reactive oxygen species (ROS), DNA damage, mitochondrial membrane potential collapse, and endoplasmic reticulum (ER) stress also contribute to aggravate DILI. Apoptosis is regarded as the terminal reaction followed by multiple signaling cascades including caspases, p53, and mitochondrial dysfunction which have been said to contribute in DILI. The SIRT1 activator is regarded as a potential candidate for DILI, because the former could inhibit signaling of p53, NF-*κ*B, and ER stress. On the other hand, overexpression of SIRT1 also enhances the activation of antioxidant responses via Kelch-like ECH-associated protein 1- (Keap1-) nuclear factor- (erythroid-derived 2-) like 2 (Nrf2) signaling. The current manuscript will highlight the mechanism of DILI and the interaction of SIRT1 with various cytoplasmic factors leading to DILI along with the summary of potent SIRT1 agonists.

## 1. Introduction

Recently, drug-induced liver injury (DILI) has gained attention in hepatology and gastroenterology [1]. About 1% or less than of DILI cases are reported to cause liver injury, but it remained a frequent cause of acute liver failure throughout the developed countries [2]. The frequent etiology of DILI is linked with herbal products, antibiotics, chemotherapeutics, and immunomodulatory agents [3]. In recent decades, research has mainly focused on finding potential agents such as antitubercular [4], acetaminophen (APAP) [5], and inorganic heavy metals [6] that cause DILI. DILI gained much attention due to its importance in drug development along with its roles in failure or withdrawal of drug development [7]. The key issue related to treating DILI is to stop using the drug instantly that is a cause of liver injury in a dose-dependent fashion [8, 9]. The epidemiological studies of DILI showed that the dosage is a major determining factor of the risk that humans suffer from idiosyncratic adverse drug reaction [10]. However, dose is not the only factor that discriminates high- from low-risk drugs, which lowers the prediction of DILI and increases the number of patients suffering DILI [11]. In addition, immune response has also played an important role in DILI [12]. Infiltration of inflammatory cells into the liver is often perceived, hinting a role for the innate immune system [13, 14]. However, there is still no effective treatment available for DILI, hence attracting huge focus for in-depth studies on the mechanisms involved in it.

The sirtuins such as SIRT1 regulate a huge number of physiological phenomena particularly energy metabolism and stress responses [15, 16]. Besides deacetylation of histones, SIRT1 is also involved in controlling DNA repair, tissue regeneration, cell survival, inflammation, neuronal signaling, and circadian rhythms [17, 18]. Dynamic changes in SIRT1 expression and activity were observed in different DILI models [19–22], which revealed its beneficial effects on APAP-induced liver injury [22–24]. Moreover, mouse livers with inactive SIRT1 have more protection against endotoxemic liver injury by acetylating and activating NF-*κ*B [25]. However, the overexpression of SIRT1 is linked with hepatocellular carcinoma cells (HCC) and tumor tissues, where it helped migration and invasion of HCC along with tumor metastasis in vivo by inducing epithelial-mesenchymal transition [26, 27]. Moreover, higher SIRT1 expression levels are linked with the total number and size of tumors [26]. Recently, a huge number of studies claimed that modulation in the expression of SIRT1 is linked with various aging-linked conditions [28].

Herein, we focused the potent effect of SIRT1 on ameliorating drug-induced liver injury (DILI), which may be a novel target for DILI treatment and other liver diseases, meanwhile shedding light on potent SIRT1 activators for further exploration of their roles in SIRT1 expression.

## 2. Drug-Induced Liver Injury (DILI)

**Figure 1:**
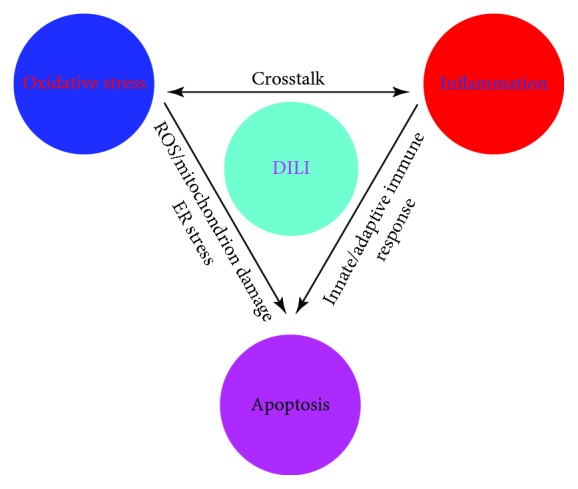
A flow chart of drug-induced liver injury (DILI) and how oxidative stress, inflammation, and apoptosis are linked with each other in DILI.

Drug-induced liver injury (DILI) is interlinked with various factors such as oxidative stress, inflammation, and apoptosis (Figure 1). DILI is classified into two subtypes according to their different traits of hepatotoxicity, i.e., intrinsic or dose-dependent DILI and idiosyncratic DILI. The former type is predictable in humans or animal models, whereas the latter type of DILI is an unpredictable injury that cannot be explained by the known pharmacological properties.

### 2.1. DILI-Caused Oxidative Stress

APAP is currently the most studied drug, which causes intrinsic DILI [29, 30], while the mechanism of APAP-induced liver injury is much clear in oxidative stress, mitochondrial dysfunction, and immune response [31–33]. APAP has higher absorption rate if administered orally, and the liver is the first site to metabolize and get damaged by various metabolites of APAP. APAP metabolism is accomplished dominantly via sulfation and glucuronidation. Another way of APAP metabolism is through cytochrome P450 (predominantly the 2E1 isoform), which metabolizes APAP into reactive metabolite N-acetyl-p-benzoquinone imine (NAPQI) by redox reactions. NAPQI leads to the depletion of glutathione (GSH) through attacking free thiols in mitochondria and cytoplasm [34]. Highly expressed NAPQI binds with proteins at sulfhydryl groups to form protein adducts [35], leading to oxidative stress and mitochondrial dysfunction, which is critical in hepatotoxicity [36]. During dysfunction of mitochondria, accumulation of great amounts of reactive oxygen species (ROS) has been reported that causes oxidative stress and activates signaling cascades (Figure 1). However, some drugs that cause DILI have a variety of ways to accumulate ROS [37].

ROS are also able to activate cytoprotective signaling in the cells such as the nuclear factor E2-related factor 2 (Nrf2)/Kelch-like ECH-associated protein 1 (Keap1)/heme oxygenase 1 (HO-1) pathway [38]. Under normal conditions, newly translated Nrf2 gets attached with Keap1 and is being degraded by p62-dependent autophagy to maintain lower levels of Nrf2 in the cytoplasm under normal condition [39]. However, when the Keap1 is oxidized or bound with NAPQI, the Nrf2 gets disassociated from Keap1 and translocated into the nucleus where it binds with the antioxidant response element (ARE) to activate antioxidant genes [40].

### 2.2. DILI-Caused Organelle Damage

The damage of organelles is another critical factor that leads to necrosis or apoptosis of hepatocytes in DILI [41, 42]. Mitochondria play an important role in supplying energy to various parts of cells, but any slight dysfunction may cause necrosis [41, 43]. The induction of mitochondrial permeability transition (MPT) enhances permeability in the mitochondrial membranes and allows the exit of molecules of variable sizes [44]. Furthermore, the collapse of mitochondrial membrane potential and the release of cell death-related proteins lead to apoptosis [45]. ROS is also contributed by the MPT pore, which in turn exaggerates oxidative stress and DNA damage, while on the contrary, *β*-oxidation and adenosine triphosphate (ATP) production are also regulated accordingly [46]. The ER stress also plays a critical role in APAP-mediated hepatotoxicity and helps synthesize proteins and their folding and secretion [47]. Once the function of ER is disturbed by overdosed APAP, its metabolites cause a severe dysfunction that leads to accumulating misfolded proteins and creating ER stress [48, 49]. The underlying mechanism of APAP-induced ER stress is not much clear yet, but various hypotheses explained the process quiet reasonably. One of the opinions is that APAP oxidizes ER oxidoreductases endoplasmic reticulum protein 72 (Erp72) and protein disulfide isomerase (PDI) of hepatocellular microsomes [50, 51]. NAPQI gets covalently attached with microsomal protein calreticulin and PDI, which have crucial roles in protein folding and create ER stress [52]. Moreover, accumulation of various ROS and disregulation in proper mitochondrial functions contribute to ER stress [53].

### 2.3. DILI-Caused Immune Responses

Various types of immune responses are also obligatory in DILI. The interaction between DILI and the immune system in both innate immune responses and adaptive immune response has a clear and detailed mechanism reported by multiple hypotheses. In innate immune response, the main hypothesis pointed out that neoantigen stimulates cells and causes inflammation by binding to scavenger receptors (SCRs), mannitol receptors (MRs), and Toll-like receptors (TLRs) in macrophages [54]. The activation of killer cells (KC) is beneficial in ameliorating APAP-induced hepatotoxicity through anti-inflammatory effects [55]. High-mobility group box 1 (HMGB1) protein is believed to activate immune cells as damage-associated molecular patterns (DAMPs) [56]. HMGB1 activates KC by releasing cytokines (TNF-*α*, interferon *γ* (IFN*γ*), and IL-1) [57, 58], while the roles of NK/NKT cells remained controversial yet, because of the secretion of cytokines (IFN*γ* and IL-4) that ameliorate liver injury [59, 60]. However, other studies argued that the significant differences exist in cytokine levels produced by NKT cell-deficient mice [61, 62]. In adaptive immune response, APAP along with its metabolites acts as haptens that bind to liver proteins. These drug-protein adducts are then processed by antigen-presenting cells (APC), and the antigen associates with major histocompatibility complex (MHC) class II molecules. After that, CD4 T-cell gets activated resulting in adaptive immune response, which then triggers CD8 cytotoxic T-cell activation leading to the expression of FasL, TNF-*α*, and other proteins that mediates cell apoptosis [63].

### 2.4. Existing Treatments of DILI

Following the recommended guidelines, three main treatments are prescribed when DILI gets diagnosed. First of them, withdrawal of the drugs or immediately discontinuation of drugs that are not indispensable for control of underlying diseases can resume liver health up to 95% or even lead to absolute recovery of the liver [64]. It is believed that drugs need to be withdrawn when the elevation of ALT or AST is <3 times of the upper limit of normal (ULN) and without clinical symptoms, even if those are not necessarily defined as liver-injuring drugs [65]. And the dosage of hepatotoxic drugs should be reduced when it is necessary in controlling other diseases and there are no other substitutable agents [65]. During pregnancy in DILI, not only the withdrawal of drugs is crucial but also the careful monitoring of the fetus is recommended or even in certain cases, the abortion can also be considered depending on the severity of DILI [65].

In the second step, the pharmacotherapy is recommended where N-Acetylcysteine (NAC) is able to clear out free radicals. NAC is recommended by ACG Clinical Guidelines published for the diagnosis and treatment of DILI and the early stage of acute liver failure (ALF) [66]. However, NAC is not recommended for children with acute liver failure [66]. Besides, magnesium isoglycyrrhizinate is recently approved to treat acute DILI by the Chinese Food and Drug Administration (CFDA), due to its efficacy of reducing the serum ALT level in randomized controlled studies [67]. Finally, at the 3^rd^ step, liver transplantation is recommended in case of severe coagulation disorders, encephalopathy, and decompensated cirrhosis [68].

## 3. SIRT1 Helps Alleviate Oxidative Stress in DILI

**Figure 2:**
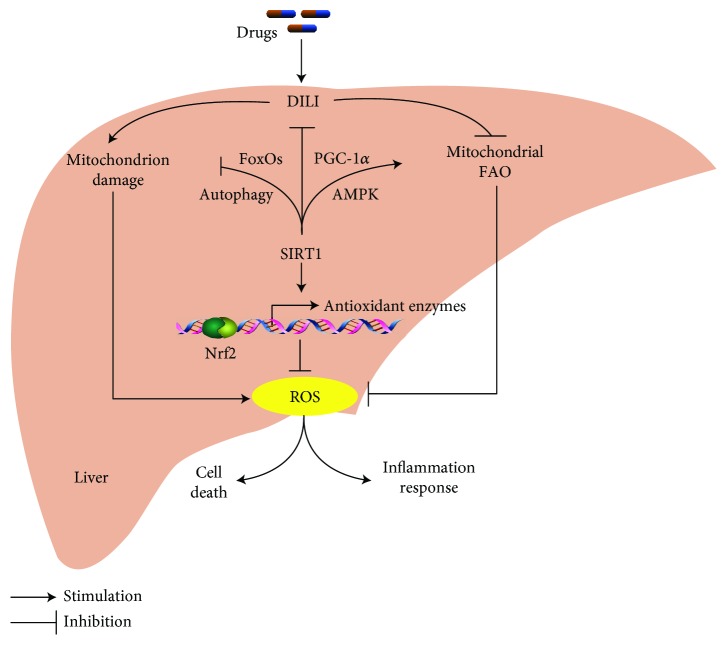
The participation of SIRT1 in protecting the liver from drug-induced liver injury (DILI). DILI results in the sharp increase of ROS (reactive oxygen species) mainly by repressing mitochondrial fatty acid oxidation (FAO) and damaging mitochondrion. Multiple signaling pathways are stimulated by SIRT1 such as Nrf2, AMPK/PGC-*α*, and FoxOs/autophagy, to exert its inhibitory effects on DILI.

DILI increases lipogenesis and ultimately the generation of ROS within cells while inhibiting mitochondrial fatty acid oxidation (FAO) (Figure 2). SIRT1 mediates responses to check lipogenesis and directly inhibits generation of ROS or alternatively via upregulation of mitochondrial FAO (Figure 2). When the individual is exposed to various harmful irritants/pollutants, a large amount of ROS and reactive nitrogen species (RNS) is produced. ROS enhances the expression of proteins and genes in the cellular antioxidant system, such as manganese superoxide dismutase (MnSOD), catalase (CAT), glutathione peroxidase 1 (GPX1), and GSH, which largely eliminates ROS. Under heavy oxidative stress, the excessively generated ROS or RNS disrupts the cellular homeostasis of oxidants and antioxidants, or in severe and highly reactive species, the tissues get damaged. SIRT1 activates various signaling cascades to alleviate oxidative stress, which is mainly reflected by the increase of lipid peroxidation and improvement of mitochondrial functions.

### 3.1. Lipid Oxidation

The ROS mediated by decreased mitochondrial FAO and increased lipogenesis play an important role in liver and other tissues [69]. Under normal conditions, both cellular oxidants and antioxidants are in the state of coordination and dynamic balance, maintaining a wide range of physiological and biochemical reactions along with various stress responses.

Excessive abuse of ethanol is a dominant factor that induces lipid deposition in the liver via depleting GSH levels and leading to ROS-mediated liver damages, which is linked with hepatic steatosis, hepatic fibrosis, and even hepatocellular carcinoma [70–72]. All this leads to two situations, i.e., either an increased level of fatty acid synthesis or a decreased level of fatty acid metabolism in the liver. AMP-activated protein kinase (AMPK) plays a key role in regulating fatty acid synthesis and metabolism [73]. Acetyl-CoA carboxylase (ACC) is a rate-limiting enzyme during fatty acid biosynthesis. Studies showed that ethanol/alcohol can inactivate AMPK, which further inhibits the phosphorylation of ACC to promote the fatty acid biosynthesis [74]. Methyl ferulic acid (MFA), a biologically active monomer extracted and purified from the Chinese herbal plant *Securidaca inappendiculata* Hasskarl, improves acute liver damage induced by ethanol closely associated with upregulation of the SIRT1 levels [75].

The peroxisome proliferator-activated receptors (PPAR-*α*, PPAR-*β*, and PPAR-*γ*) are a group of nuclear receptor proteins that function as transcription factors regulating the expression of genes involved in multiple cellular metabolisms, especially in energy metabolism. PPAR-*α* is expressed in the liver cells and helps promote oxidation of lipids. Carnitine palmitoyl-transferase 1A (CPT1A) is a rate-limiting enzyme taking part in *β*-oxidation of fatty acids [76]. Ethanol can inhibit the activation of PPAR-*α* and CPT1, which weakens the lipid oxidation and leads to the lipid deposition in the livers [77]. SIRT1 activation can increase the level of PPAR-*α* and peroxisome proliferator-activated receptor-*γ* coactivator 1*α* (PGC-1*α*) [78], which is the coactivator of PPAR-*α* to exhibit the protective effects during liver injury [79]. MFA has a positive effect on ethanol-induced hepatic steatosis by increasing the levels of AMPK, FoxO1, SIRT1, PPAR-*α*, and CPT-1A. PPAR-*γ* is well stated to take part in the promotion of the biosynthesis of lipids within the liver [80]. Moreover, PPAR-*γ* is repressed by SIRT1 to promote lipogenesis [81]. Activation of SIRT1, on the one hand, increases FAO expression by stimulating the PPAR-*α*/PGC-1*α* axis and decreases lipogenesis by targeting PPAR-*γ*, leading to amelioration of lipid metabolism and ultimately improvement of DILI. Consistent with this, isoniazid-rifampicin-induced liver injury was successfully ameliorated by upregulation of the expression of SIRT1 [79].

In addition, SIRT1 and AMPK combined and work in the fatty acid metabolism. It is further said that thymoquinone (TQ) activates AMPK to reduce the alcohol-mediated liver injury by upregulating SIRT1 [82]. Alcohol consumption alters lipid homeostasis particularly by decreasing PPAR expression and increased activation of sterol regulatory element-binding proteins (SREBP-1) via an AMPK-dependent way [83]. Some evidences showed that SIRT1 was able to stimulate AMPK activation via regulating liver kinase B-1 (LKB1) which is a major serine/threonine kinase which binds closely with AMPK to direct the activation of the downstream kinases [84]. It has also been stated that SIRT1 can inhibit the expression of SREBP-1 and fatty acid synthase (FASN), which is the well-known mechanism of resveratrol's protective effect on high-fructose corn syrup-induced hepatic dysfunction [85].

SIRT1/PGC-1*α* cascade may act as upstream of the Nrf2 signaling pathway to alleviate DILI. Upon activation, Nrf2 translocated into the nucleus where it binds with the antioxidant response element (ARE) and activates antioxidant genes. Nfr2 intentionally activates HMOX1 that translated into HO-1 and helps express NAD(P)H quinone dehydrogenase 1 (NQO1) and the glutamate-cysteine ligase catalytic/modifier subunit (GCLC/GCLM). NQO1 is the regulator of lipid metabolism, while HO-1 actively metabolizes heme to scavenge free radicals in the cytoplasm. GCLC and GCLM regulate the cellular redox status to remove ROS quite efficiently [86]. Furthermore, Nrf2 plays a critical role in transcriptional upregulation of ATP-binding cassette (ABC) transporters essential for cellular defense in response to oxidative stress [87]. SIRT1 transforms the free fatty acids into glucose by acetylation and activation of PGC-1*α* and FoxO1 in short-term fasting, along with the increase in Nrf2 transcription and activation [88]. Fasting can induce the accumulation of cAMP, but cAMP/PKA and SIRT1 are the upstream regulatory factors that rapidly activate Nrf2-ABC transporters, which help to clear various chemicals and biliary excretions in the liver cells in response to chemical stimulants and liver injury [86, 88].

### 3.2. Mitochondrial Function

During oxidative stress, reduction in mitochondrial membrane potential (MMP) due to excessive ROS and mitochondrial permeability transition pores (MPTP) are two major factors causing mitochondrial damage. The interactions between excessive ROS and the hepatic mitochondrial membranes are major indicators under oxidative stress. Certain studies have found that D-galactosamine/lipopolysaccharide- (D-GalN/LPS-) induced acute liver injury in mouse models has a higher production level of malondialdehyde (MDA) [89]. An end product of lipid hydroperoxide (LPO) may lead to decreased mitochondrial membrane fluidity, even under severe damages [90]. For this kind of mitochondrial membrane damage or lipid peroxidation caused by excessive ROS, one of the effective ways is to enhance the activation of the cellular antioxidant system to eliminate heavy burst of ROS. It is further said that mitochondrial antioxidant defense was enhanced by curcumin when challenged with D-GalN/LPS [21]. Curcumin is a chain-breaking antioxidant which is a lipophilic substance that can be incorporated into the biofilms that directly protect cells from ROS. Curcumin modulated the mRNA expression of SIRT1 in liver cells that regulates the activity of FoxO3 and alters the expression of MnSOD and Cat [16, 21].

Mitochondrial permeability transition pores (MPTP) play a vital role in maintaining mitochondrial physiology and performance. A sharp rise in ROS generation leads to the opening of MPTP, resulting in the imbalance of H^+^ on the inner membrane of mitochondria, destroys membrane proteins, inhibits ATP synthesis, and causes mitochondrial swelling, all of which may exacerbate necrotic or apoptotic cascades leading to rapid cell death [91]. Resveratrol is able to reinstate SIRT1 activation which resists against oxidative stress through the upregulation of antioxidants such as superoxide dismutase 2 (SOD2), which inhibits the mitochondrial injury by swapping out excessively generated ROS [92]. It has also been found that cyclosporine A (CsA) can inhibit MPTP constitution protein cyclophilin D to protect mitochondrial functional integrity under severe shocks [93].

An enhancement in mitochondrial biogenesis is also an important way to improve mitochondrial functionality. For instance, cadmium (Cd) causes mitochondrial swelling and checks mitochondrial activity including oxidant capacity and ATP synthesis [94]. By acting on melatonin receptor 1 (MTR1), melatonin activates the SIRT1/PGC-1 signaling pathway, allowing SIRT1 to interact with PGC-1 in deacetylation, thus improving the activation of PGC-1. Moreover, it can accelerate mitochondrial biogenesis and maintain mitochondrial physiology [95]. In addition to it, AMPK is situated at the upstream of the SIRT1 pathway and Cd treatment leads to a significant increase in the pAMPK protein level but melatonin does not change AMPK signaling [96].

## 4. SIRT1 Decreases the Inflammatory Responses in DILI

**Figure 3:**
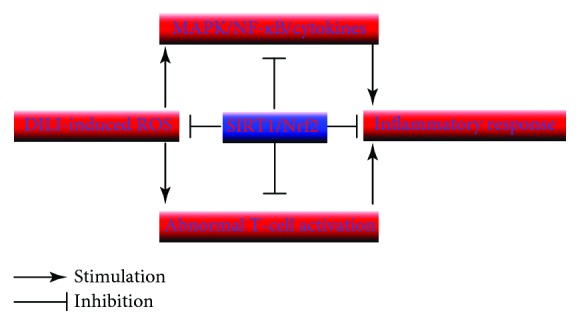
The role of DILI-induced ROS that activates inflammatory factors and the role of SIRT1 along with Nrf2 for inhibition of inflammatory responses in the liver.

There are numerous types of immunocytes that initiate inflammatory reactions in the liver including macrophage Kupffer cells (KCs), T-cells, B-cells, and natural killer (NK) cells. All these inflammatory responders play an important role in the drug-induced liver injury. Heavy ROS activates a variety of inflammatory factors, but SIRT1 along with Nrf2 efficiently inhibits these activated inflammatory responses in the liver cells (Figure 3). The mechanism of inflammation is elucidated mainly in the NF-*κ*B signaling pathway and various SIRT1 activity-related agents like HMGB1 and SRT1720.

### 4.1. NF-*κ*B Signaling

NF-*κ*B is a dimer protein that is generally composed of two functional subunits (p65 and p50) and binds to its natural inhibitory protein I*κ*B, which can prevent NF-*κ*B from nuclear translocation and regulation of related target genes. Once the hepatocyte gets stimulated, the Toll-like receptor 4 (TLR4)/myeloid differentiation factor 88 (MyD88) receptor receives the stimulant that further activates the three subunits of the MPAK (p38, JNK, and ERK) signaling pathway. The regulation of proinflammatory cytokines has significant contribution in activation of the NF-*κ*B pathway. Various compounds particularly ethanol [97], carbon tetrachloride (CCl_4_) [98], APAP [22], and mercuric chloride [86] help to release numerous proinflammatory cytokines and subsequently cause inflammations.

In ethanol-induced hepatic injury, lipid peroxidation adducts such as 4-HNE and MDA are formed which aid elimination of GSH and accumulation of TG and enhanced the release of TNF-*α*, TGF-*β*, and IL-6 [99]. These various cytokines can activate the MPAK and NF-*κ*B signaling cascades. Moreover, the plant extracts of *Ulmus davidiana* var. *japonica* (RUE) treatment can inhibit the activation of MAPKs and NF-*κ*B signaling, reducing the expression of IL-6, IL-1*β*, and IL-18 and the downstream targets of NF-*κ*B signaling cascades. Moreover, RUE increases the expression of SIRT1 and ultimately stimulated the activation of AMPK-*α* and increased the expression of PGC-1*α*, thus reducing the fatty acid oxidation [97].

The higher ROS production can result in hepatocyte damage, and persistent accumulation of ROS leads to inflammation and release of TNF-*α* [100]. In HgCl_2_-induced liver injury, Hg^2+^ gets tightly complexed with hydrosulphonyl moieties and depletes intracellular hydrosulphonyl moieties and leading to the generation of more ROS [86]. TNF-*α* binds to tumor necrosis factor receptors (TNFR) to form a complex that binds with TNFR type 1-associated death domain protein (TRADD) and to activate p38 MAPK and NF-*κ*B [101]. It was also suggested that luteolin reduces activation of NF-*κ*B and phosphorylation of p38 under HgCl_2_-induced injury. Additionally, luteolin also activates SIRT1 which is inhibited by HgCl_2_. SIRT1 directly reduces NF-*κ*B and p53 activation via promoting deacetylation of NF-*κ*B at p65 and p53subunits [102, 103]. Furthermore, SIRT1 activates the Nrf2/Keap1 pathway to decrease ROS generation and their accumulation in the cytoplasm [88]. Hence, luteolin-mediated SIRT1 plays an important function in regulating inflammation, apoptosis, and antioxidant defense systems in HgCl_2_-induced hepatotoxicity.

Immune response is also modulated by protective effects of SIRT1 during DILI. It is revealed that concanavalin A (ConA) promotes the release of proinflammatory cytokines (TNF-*α*, IFN*γ*) which lead to liver injury [104]. Recently, SIRT1 expression at transcriptional and translational levels was increased in activated T-cells and knockout of SIRT1 resulted in an abnormal increase of T-cell activation and lowered the tolerance of CD4^+^ T-cells [105]. An abnormal increase in T-cell activation and defects in innate immune responses to microbes were clear in patients with acute liver failures [63]. With T-cells being activated overly by ConA, the inflammatory responses can lead to hepatitis or in certain cases resulted in autoimmune diseases. Treatment with salvianolic acid A (SalA) enhanced the expression of SIRT1, which might be used to restrict the abnormal T-cell activation restoring antimicrobial responses in patients with ALF. On the contrary, SalA is also able to negatively regulate NF-*κ*B-dependent inflammatory cascades by inhibiting IKK*β*. It is clear that the protective impact of SalA has an important part in ConA-induced inflammations.

In the NF-*κ*B signaling, instead of the upregulation of expression of proinflammatory cytokines, the regulation of TLR/MyD88 is also an important factor that negatively affects inflammations [101]. Downregulation of TLR4 reduces NF-*κ*B activation, while dimethylnitrosamine (DMN) causes oxidative stress to produce superfluous ROS, which is the igniting agent of inflammation [106]. Moreover, DMN is able to aid in releasing proinflammatory cytokines (TNF-*α*, IL-1*β*, and IL-6) and further aggravate the activation of NF-*κ*B signaling. Meanwhile, NF-*κ*B activation modulates iNOS that leads to the production of NO and causes inflammation and necrosis. Dioscin inhibits expression of TLR4, MyD88, p50, p65, TNF-*α*, IL-1*β*, IL-6, iNOS, and NO at transcriptional levels and effectively improves the DMN-induced acute liver injury [106].

Trace elements potentially target the modulation of NF-*κ*B. In CCl4-induced liver injury, excessive ROS may likely activate Kupffer cells and help releasing proinflammatory cytokines. Grape seed oil (GSO) can upregulate the gene expression of SIRT1 to give protection by downregulating NF-*κ*B. Interestingly, these trace elements can be the cofactors for many enzymes to perform their roles in SODs (CuZnSOD and MnSOD) and are involved in oxidative stress and possible inflammations [107].

### 4.2. Other Factors

High-mobility group protein box 1 (HMGB1) is reported to play an important role in sepsis, and it can activate innate immune cells to give rise to antigen-presenting cells [56]. Under normal condition, HMGB1 resides in the nucleus that remained bound with DNA to stabilize nucleosomes and facilitates mRNA expression at transcriptional levels [82]. Extracellular HMGB1 release can cause inflammation and stimulate innate immune cell migration and activation via nucleocytoplasmic HMGB1 translocation. Acetylation is important for activated HMGB1 to release from the nucleus to the cytoplasm. SIRT1 may regulate HMGB1 translocation via deacetylation [108]. Resveratrol can upregulate SIRT1 to inhibit inflammation and hence attenuated severe liver injury following sepsis [109]. Moreover, SRT1720 reduces the release of inflammatory cytokines (TNF-*α*, IL-6) to block the inflammatory reactions [110].

## 5. SIRT 1 Alleviates Apoptosis in DILI

Apoptosis ends up with the DNA fragmentation which can intensify oxidative stress along with inflammation and leads to irreversible cell death adopting the apoptotic pathway or necrosis [107]. In the process of apoptosis, upstream factors of apoptosis have interaction with related receptors (TNFR1/2, Fas, and DR3/4/5) and further activate the apoptotic signaling pathways, which can be concluded into TNF/TNFR, Fas/FasL, and mitochondrion-mediated and endoplasmic reticulum-mediated pathways. Then the apoptotic genes get activated and DNase or caspases will execute apoptosis to induce DNA fragmentation.

### 5.1. The Mitochondrion-Mediated Signaling Pathway

The mitochondrial membrane integrity is essential for ethanol-induced cell death [111]. When the apoptosis fragments stimulate the hepatocyte, cytochrome C (CytC) and the apoptosis-inducing factor (AIF) restricted to the mitochondria are released into the cytosol. CytC triggers caspase-3 and executes the caspase series, which can directly result in apoptosis. AIF, which is a proapoptotic protein, is independent on the caspase pathway and directly induces apoptotic cell death. According to the study, ethanol treatment enhances the release of CytC and AIF, thus promoting apoptosis and subsequent liver injury. However, carnosic acid (CA) can improve the ethanol-induced liver injury via the SIRT1/p66Shc pathway [112]. CA enhances SIRT1 expression which is being inhibited by ethanol treatment. It is known that SIRT1 is a crucial factor in the amelioration of ethanol-induced mitochondrial damage under heavy oxidative stress. Growing studies have indicated that SIRT1 can restrain the expression of p66Shc at transcriptional levels by decreasing acetylation of p66Shc promoter-bound histone H3 [112]. Moreover, p66Shc suppresses its interaction with mitochondrial CytC to reduce apoptosis [113]. Similarly, SIRT1 has a similar effect on AIF. CA can significantly attenuate ethanol-induced liver injury through a SIRT1/p66Shc-mediated mitochondrial pathway. In addition, it has shown that SalA can decrease the expression of caspase-3 and p66Shc and increase the expression of Bcl-xL, which has been well documented that SalA has a protective effect on the concanavalin A- (ConA-) induced hepatocyte apoptosis. The result has shown that pretreatment of SalA augmented SIRT1 expression and inhibited p66Shc expression to reverse the upregulation of p66Shc expression by ConA [104]. Moreover, the Bcl-2 family can inhibit apoptosis while Bak promotes cell apoptosis in the mitochondrial signaling pathway [106].

### 5.2. DNA Damage and p53 Pathway

Not only the excessive ROS but toxic agents (including CCl_4_ [98], APAP [22], and cadmium [94]) and UV radiation [107] could lead to DNA damage and ultimately apoptosis. CCl_4_ increases the iNOS level releasing NO into the cells, which combines with superoxide ions to form peroxynitrite and metabolically active trichloromethyl radicals that results in either direct or indirect DNA damage to promote apoptosis [107]. Furthermore, the expression of NO by iNOS can also be regulated by the activation of NF-*κ*B, mainly by oxidative stress, and CCl_4_ can indirectly upregulate the series of caspases, the dominant players in apoptosis. GSO protected CCl_4_-induced liver injury via inhibiting caspase-3 activation, through activation of SIRT1, which blocks the expression of NF-*κ*B and decreases the production of NO [107].

On the other hand, p53 is the sensor of DNA damage and cell death and it promotes apoptosis by regulating the levels of caspase-9, caspase-3, and Bcl-2 family members [114]. Few studies claimed that dioscin has a positive effect on DMN-induced acute liver injury and inhibitory effect on hepatocyte apoptosis by activating SIRT1 [106, 115]. Interferon regulatory factor 9 (IRF9) plays a key role in inducing apoptosis by decreasing the SIRT1 expression level and increasing p53 expression [106, 116]. In addition to it, dioscin significantly decreased the levels of IRF9, p53, Bax, caspase-3, caspase-9, Fas, and FasL, while it increased the levels of SIRT1 and Bcl-2 [106]. Therefore, dioscin can be a quite efficient protector to improve the cell apoptosis and DMN-induced acute liver injury.

In addition, it has been illuminated that the MAPK-p53 axis is a major signaling pathway involved in the apoptosis of hepatocytes. Furthermore, the activation of p38 MAPK can regulate p53 transcriptional activation and translocation into the nucleus [117]. Similarly, the increased level of MAPK p38 can also induce excessive ROS production and further modulates p53-mediated apoptosis in human hepatocytes [97]. RUE was reported to negatively regulate MAPK activity and downregulate p53 expression blocking apoptosis in chronic alcohol-mediated liver injury. Moreover, pterostilbene (Pte), a natural dimethylated analog of resveratrol from blueberries, alleviates sepsis-induced liver injury by reducing the expression of acetylated (Ac) FoxO1, Ac-p53, and p38MAPK activities and the potential mechanism is associated with SIRT1 signaling activation [118, 119].

### 5.3. ER Stress

ER, which is closely associated with DILI, also contributes to apoptosis in intrinsic and extrinsic pathways. Through the intrinsic pathway, Ca^2+^ was released from ER lumen by ER-localized Bak, resulting from the conformational changes and oligomerization of Bak and Bax at the ER membrane [120]. Disruption of the Ca^2+^ pool activates calpain in the cytosol and converts procaspase-12 to caspase-12 thus activating caspase-9 and caspase-3 to approach apoptosis [121]. On the other hand, the released Ca^2+^ is taken up by mitochondria, leading to collapse of inner membrane potential and subsequently the initiation of apoptosis [122]. On the other hand, in the extrinsic pathway, IRE forms the complex with TNF receptor-associated factor 2 (TRAF2) and apoptosis signal-regulating kinase 1 (ASK1) to activate JNK [123]. Han et al. reported that reduced silent information regulator 1 signaling exacerbates sepsis-induced ER stress in sepsis-induced myocardial injury [124]. ER stress has recently emerged as major regulators in drug-induced liver injury [49]. Further experiments are needed to confirm whether SIRT1 is involved in ER stress in DILLI.

### 5.4. TNF/TRAF Pathway

TNF-*α* is the key factor that mediates the association between oxidative stress and NF-*κ*B. NF-*κ*B activates a series of genes such as c-IAP1 and c-IAP2 to inhibit TNF-*α*-induced apoptosis by suppressing the activity of caspases [125, 126]. IAPs inhibit CytC-induced proteolytic processing of caspase-3. However, the recruitment of c-IAP1 and c-IAP2 in response to TNF-*α*, which is dependent on the interaction with TRAF1 or TRAF2, is indispensable to inhibit apical caspase-8 [125]. Multiple studies showed that SIRT1 signaling activation attenuates DILI by reducing the TNF/TRAF pathway and finally inhibits hepatic apoptosis [118, 127, 128].

### 5.5. Other Mechanisms and Connections

Meanwhile, certain mechanisms get interacted with each other in DILI. SIRT1 is also the activator of autophagy in DILI and provides potential protection against hepatotoxicity. Many of the in vivo studies depicted autophagy as an important mechanism which involves lysosomal degradation to get rid of the damaged cellular inclusions in order to maintain homeostasis particularly in hepatocytes [129]. In continuation, autophagy clears excessive lipids/fat molecules to ameliorate hepatocyte steatosis [130]. In SIRT1-mediated autophagy, FoxO3 poses a crucial part in the forming of amplification of stress-linked autophagy. Overexpression of the negative form of FoxO3a abolishes the induction of ethanol-induced autophagy [131]. Resveratrol activates SIRT1, and the expression of autophagy-linked genes was improved mainly because of the deacetylation by SIRT1. Furthermore, FoxO3a^−/−^ mice were treated with ethanol exhibiting decreased expression of autophagy-related genes [131]. This manuscript suggested the importance of FoxO3a in SIRT1-linked autophagy. Besides, acetylate FoxO1 was hinted to promote autophagy by binding to Atg7 in cancer cells [23]. The FoxO family has diverse biological functions including cell cycle, apoptosis, oxidative stress, DNA damage repair, and glucose metabolism; thus, attention is needed to be paid in the SIRT1-autophagy activation process [131].

Self-protection mechanism such as regeneration is also involved in the SIRT1-activated DILI attenuation. The SIRT1 agonist, resveratrol, was applied as pretreatment before APAP administration. SIRT1 activation represses p53 signaling, thus inducing cell proliferation-associated proteins such as CDK4, PCNA, and cyclin D1 to promote proliferation of hepatocytes [79].

**Figure 4:**
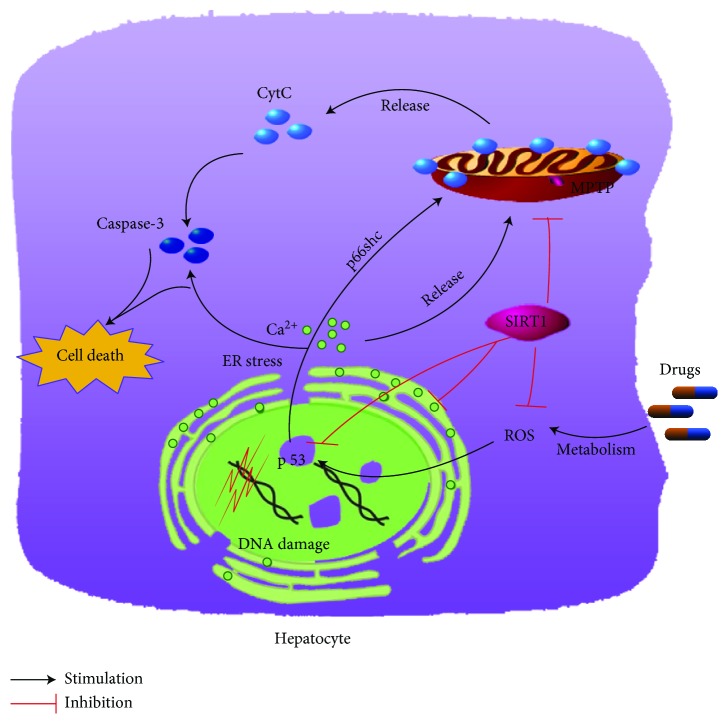
Effect of SIRT1 on maintaining cellular physiology in DILI. By preventing the generation of reactive oxygen species (ROS), endoplasmic reticulum (ER) stress, and mitochondria dysfunction, SIRT1 blocks the damage induced by p66Shc, Ca^2+^, and cytochrome C (CytC).

It is now clear to say that SIRT1 plays a key role in ameliorating DILI by inhibiting oxidative stress, inflammation, and apoptosis by interacting with other signaling pathways, including p53, HMGB1, and autophagy (Figure 4). In DILI, oxidative stress-causing agents also contribute to apoptosis. The efflux of cellular ROS can attack DNA to create DNA lesions and finally lead to apoptosis or necrosis in the cells [37]. These DNA fragments can aggravate the oxidative stress in return. Inflammation can also be developed into apoptosis, and both mechanisms are regulated by the p38 MAPK pathway. The p38 can significantly stimulate p53 function, which plays a critical role in the apoptosis induction, while p38-MAPK can promote phosphorylation of I*κ*B (inhibitor of NF-*κ*B) which dissociates with NF-*κ*B to induce inflammation. There are some reliable evidences that showed that NF-*κ*B can be suppressed by luteolin and decrease the relative protein levels of Bax and increase the Bcl-2 levels, which can inhibit cell apoptosis [86].

There is also a close relation between oxidative stress and inflammation. Nrf2 has a negative effect on TNF-*α* expression, playing a crucial role during oxidative stress and inducing the production of the downstream target proteins of Nrf2 such as NQO1, HO-1, and SOD [132]. Therefore, we conclude that Nrf2 is a key regulator in antioxidant and anti-inflammatory defense systems.

## 6. Outlook

Over the last several decades, investigators continued to broaden our knowledge in understanding the intracellular signaling mechanisms leading to DILI in hepatocytes in experimental animals and humans. However, DILI is still a critical problem that plagues individuals across the world, largely due to the increased consumption of drugs and the lack of specific therapies and antidotes. Recent studies highlighted the therapeutic roles of SIRT1 in treating DILI. Therefore, clinical application of SIRT1 activators (Table 1) is the putative therapeutic approach against DILI. However, despite the promising findings from *in vitro* and *in vivo* studies, supporting evidence from clinical studies is lacking. Furthermore, the application of powerful genomic, proteomic, transcriptomic, and metabonomic technologies holds promise to connect SIRT1 to the established mechanisms that will enhance our understanding of the SIRT1 role in DILI. In-depth insight into the therapeutic role of SIRT1 with a goal toward modification and prevention may be fruitful in light of medical treatment of DILI limited by the deficiency of specific therapies and antidotes currently available.

## Figures and Tables

**Table 1:** Compounds that alleviate drug-induced hepatotoxicity.

Compounds		Toxic		Mechanisms	Ref.
Name	Dosage	Name	Dosage		
Dioscin	80, 40, and 20 mg/kg (mice) 60, 30, and 15 mg/kg (rats)	DMN	14 mg/mg (mice) 10 mg/mg (rats)	SIRT1↑, Nrf2↑, and Bcl-2↑ p53↓, Fas↓, and FasL↓	[99]
GSO	3.7 g/kg (rats)	CCl_4_	1 ml/kg (rats)	SIRT1↑ NF-*κ*B↓, CYP2E1↓	[100]
Luteolin	100 mg/kg (mice)	HgCl_2_	4 mg/kg (mice)	SIRT1↑, Nrf2↑ p38↓, TNF-*α*↓	[72]
SalB	15, 30 mg/kg (rats)	Ethanol	6 g/kg (rats)	SIRT1↑ p53↓, NF-*κ*B↓	[96]
RUE	100 mg/kg (mice)	Ethanol	1 g/kg (mice)	SIRT1↑, AMPK↑ p65↓, SREBP-1↓	[89]
MFA	5, 10, and 10 mg/kg (rats)	Ethanol	5% (*w*/*v*, rats)	SIRT1↑, PPAR-*α*↑ MAPK↓, p-ACC↓	[67]
TQ	20, 40 mg/kg (mice)	Ethanol	5 g/kg (mice)	SIRT1↑, AMPK↑	[74]
CA	20, 40 mg/kg (rats)	Ethanol	6 g/kg (rats)	SIRT1↑ p66Shc↓	[105]
Resveratrol	500 mg/kg (Rats)	HFCS	20% (*w*/*v*, rats)	SIRT1↑, IRS-1↑ iNOS↓	[77]
Quercetin SRT1720 Resveratrol	50 mg/kg (rats) 5 mg/kg (rats) 2.3 mg/kg (rats)	D-GalN LPS	400 mg/kg (rats) 10 *μ*g/kg (rats)	SIRT1↑, HO-1↑ Bilirubin↓	[35]
Melatonin	0.5 *μ*M (HepG2)	Cadmium	2.5, 5, and 10 *μ*M (HepG2)	SIRT1↑ Ac-PGC-1*α*↓	[88]
STR1720	10, 20 mg/kg (mice)	EE	10 mg/kg (mice)	SIRT1↑, FXR/HNF1*α*↑ IL-6↓, TNF-*α*↓	[103]
SalA	15, 25 mg/kg (mice)	ConA	18 mg/kg (mice)	SIRT1↑ p66Shc↓	[97]
Resveratrol	25, 50, and 100 mg/kg (mice)	APAP	400 mg/kg (mice)	SIRT1↑, cyclin D1↑ p53↓, CYP2E1↓	[23]
Resveratrol CAY10591	30 mg/kg (rats) 0.5 mg/kg (rats)	APAP	1 g/kg (rats)	SIRT1↑	[24]
Resveratrol	100 mg/kg (mice)	INH RIF	50 mg/kg (mice) 100 mg/kg (mice)	SIRT1↑ PPAR-*γ*↓	[71]

DMN: dimethylnitrosamine; CCl_4_: carbon tetrachloride; GSO: grape seed oil; MFA: methyl ferulic acid; EE: 17*α*-ethinylestradiol; RUE: root bark of *Ulmus davidiana* var. *japonica*; D-GalN: D-galactosamine; LPS: lipopolysaccharide; TQ: thymoquinone; CA: carnosic acid; HFCS: high-fructose corn syrup; CLP: cecal ligation and puncture; SalB: salvianolic acid B; SalA: salvianolic acid A; ConA: concanavalin; Ref.: reference.

## Supplementary Materials

Supplementary Materials SIRT1 plays a vital role in drug-induced liver injury. SIRT1 can regulate the levels of inflammation and oxidative stress, which are the main damages caused by DILI. During DILI, the repressed SIRT1 increases the immune response and oxidative damage and ultimately leads to the death of hepatocyte, in the form of apoptosis, namely, programmed cell death, or cell necrosis, both of which are the cause of liver injury. On the other hand, enhancing SIRT1 expression or activity of SIRT1 protects the liver against drug-induced injury. SIRT1 alleviates oxidative stress by targeting AMPK, Nrf2, PGC-1*α*, and FoxOs and improves mitochondrial function. The mechanism of inflammation is elucidated mainly in the NF-*κ*B signaling pathway which is tightly regulated by SIRT1.
