# Supplementary material for: The Beneficial Roles of SIRT1 in Drug-Induced Liver Injury
Source: Oxid Med Cell Longev. 2019 Jul 1;2019:8506195. doi: 10.1155/2019/8506195 (PMC6636535; doi:10.1155/2019/8506195)
Supplement: Supplementary Materials — SIRT1 plays a vital role in drug-induced liver injury. SIRT1 can regulate the levels of inflammation and oxidative stress, which are the main damages caused by DILI. During DILI, the repressed SIRT1 increases the immune response and oxidative damage and ultimately leads to the death of hepatocyte, in the form of apoptosis, namely, programmed cell death, or cell necrosis, both of which are the cause of liver injury. On the other hand, enhancing SIRT1 expression or activity of SIRT1 protects the liver against drug-induced injury. SIRT1 alleviates oxidative stress by targeting AMPK, Nrf2, PGC-1α, and FoxOs and improves mitochondrial function. The mechanism of inflammation is elucidated mainly in the NF-κB signaling pathway which is tightly regulated by SIRT1. [file 8506195.f1.pdf]

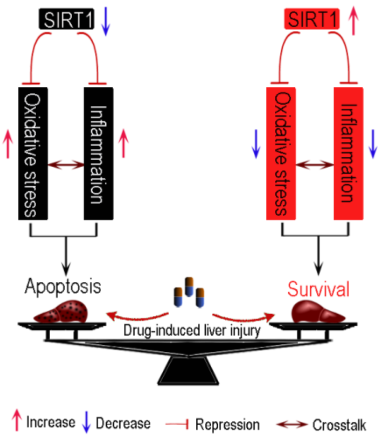

SIRT1 plays a vital role in drug-induced liver injury. SIRT1 can regulate the level of inflammation and oxidative stress, which are the main damages caused by DILI. During DILI, the repressed SIRT1 increases the immune response and oxidative damage, and ultimately leading to the death of hepatocyte, in form of apoptosis, namely programmed cell death, or cell necrosis, both of which are the cause of liver injury. On the other hand, enhancing SIRT1 expression or activity of SIRT1 protects liver against drug-induced injury. Sirt1 alleviates oxidative stress by targeting AMPK, Nrf2, PGC-1α and FoxOs and improves mitochondrial function. The mechanism of inflammation is elucidated mainly in NF-κB signaling pathway which tightly regulated by SIRT1.
